# Hankel determinant for certain new classes of analytic functions associated the activation functions

**DOI:** 10.1016/j.heliyon.2023.e21449

**Published:** 2023-10-27

**Authors:** YueJuan Sun, Muhammad Arif, Khalil Ullah, Lei Shi, Muhammad Imran Faisal

**Affiliations:** a School of Mathematics and Statistics, Shangqiu Normal University, Shangqiu 476000, Henan, P.R. China; b Department of Mathematics, Abdul Wali Khan University Mardan, Mardan 23200, Pakistan; c School of Mathematics and Statistics, Anyang Normal University, Anyang 455002, Henan, P.R. China; d Department of Mathematics, Taibah University, Universities Road, PO Box: 344, Medina, Saudi Arabia

**Keywords:** 30C45, 30C80, Hankel determinant, Activation functions, Univalent mappings, Coefficient bounds

## Abstract

In this article, we introduce two new classes of analytic functions $J_{tanh}$ and $J_{SG}$ which are associated with the activation functions and defined by the ratio of analytic representations of convex and starlike functions. For functions in these classes, our primary goal is to determine the sharp upper bounds of second and third Hankel determinants.

## 1 Introduction

We must first provide a few essential concepts to understand the fundamental terminology used throughout our major results. Let A represent the family of functions analytic in the open unit disc D={z∈C:|z|<1} and normalized by the form of

(1.1) $$f(z)=z+\sum_{l=2}^{\infty }a_{l}z^{l}$$

using the series representation. Let us denote S⊆A the family of univalent functions in D. Suppose that P contains all the functions *ϱ* which are analytic in D, ℜ(ϱ(z))>0 and

$$\varrho \left(z\right)=1+\sum_{l=1}^{\infty }c_{l}z^{l},\;z\in D.$$

For ϱ∈P, it is known as Carathéodory function [1]. A function f∈A is said to be in the class $S^{⁎}$ of starlike functions, if and only if

$$ℜ\left(\frac{zf^{\prime }\left(z\right)}{f\left(z\right)}\right)>0,\;z\in D.$$

Another significant subclass of univalent functions is the family C of convex functions, which are consisting of functions *f* defined by the analytic representation

$$ℜ\left(1+\frac{zf^{{}^{\prime \prime }}\left(z\right)}{f^{\prime }\left(z\right)}\right)>0,\;z\in D.$$

For these two subclasses, it is noted that f∈C if and only if $zf^{\prime }\in S^{⁎}$, see [2] for more details.

It is said that *g* is subordinate to *h* (written as g≺h), if there exists an analytic function *w* with w(0)=0 and |w(z)|<1 in the way of g(z)=h(w(z)) in D. If *h* is univalent in D, then g(0)=h(0) and g(D)⊆h(D).

In 1992, Ma and Minda [3] gave the class $S^{⁎}(\varpi )$ defined by

$$S^{⁎}(\varpi ):=\left\{f\in A:\frac{zf^{\prime }(z)}{f(z)}≺\varpi (z)\right\}.$$

Here, *ϖ* is a more general analytic function with ℜ{ϖ(z)}>0(z∈D) and normalized by the condition ϖ(0)=1 and $\varpi^{\prime }(0)>0$.

The Hankel determinants ([4], [5]) $H_{\lambda ,n}\left(f\right)$ (n,λ∈N={1,2,…}) admitting entries of coefficients for f∈S is given by

$$H_{\lambda ,n}\left(f\right)=\left|\begin{array}{cccc} a_{n}\; & a_{n+1}\; & \ldots \; & a_{n+\lambda -1}\\ a_{n+1}\; & a_{n+2}\; & \ldots \; & a_{n+\lambda }\\ \vdots \; & \vdots \; & \ldots \; & \vdots \\ a_{n+\lambda -1}\; & a_{n+\lambda }\; & \ldots \; & a_{n+2\lambda -2} \end{array}\right|.$$

This determinant is extensively used in both pure mathematics and technological applications, see [6], [7], [8], [9]. By restricting *f* in certain subclasses of univalent functions, the upper bounds of this determinant is well studied. For instance, Kowalczyk et al. [10] proved that $\left|H_{3,1}\left(f\right)\right|\leq \frac{4}{9}$ supposed that *f* is a starlike function. It is not an easy thing and it can be seen that there are many works attempting to solve this problem, see [11], [12]. On some recently obtained sharp bounds of third Hankel determinant for the Ma-Minda class, we list them in Table 1 for references. For bounds of this determinant on other classes of univalent functions, we refer to [18], [19], [20], [21].

**Table 1:** Results on third Hankel determinant.

Author/s	*ϖ*(*z*)	Bounds	Year	Reference
B. Rath et al.	$\frac{1}{1-z}$	1/9	2022	[13]
S. Banga and S.S. Kumar	$\sqrt{1+z}$	1/36	2020	[14]
K. Ullah et al.	1+tanh⁡(z)	1/9	2021	[15]
Shi et al.	1+sin⁡(z)	1/9	2022	[16]
Riaz et al.	$\frac{2}{1+e^{-z}}$	1/36	2022	[17]

In [22], Silverman introduced a new subfamily of A utilizing the quotient of $\left(1+\frac{zf^{{}^{\prime \prime }}\left(z\right)}{f^{\prime }\left(z\right)}\right)/\frac{zf^{\prime }\left(z\right)}{f\left(z\right)}$. Let b∈(0,1]. The considered class $J_{b}$ was given as

$$J_{b}:=\left\{f\in A:\left|\frac{1+\frac{zf^{{}^{\prime \prime }}\left(z\right)}{f^{\prime }\left(z\right)}}{\frac{zf^{\prime }\left(z\right)}{f\left(z\right)}}-1\right|<b,\;z\in D\right\}.$$

If we represent it in the form of subordination, it can be also written as

$$J_{b}=\left\{f\in A:\frac{1+\frac{zf^{{}^{\prime \prime }}\left(z\right)}{f^{\prime }\left(z\right)}}{\frac{zf^{\prime }\left(z\right)}{f\left(z\right)}}≺1+bz,\;z\in D\right\}.$$

In [23], the non-sharp bounds on third Hankel determinants for $f\in J_{b}$ were calculated. Recently, many interesting properties on the classes of analytic functions satisfying some conditions given by the ratio of convex functions and starlike were investigated, we refer to [24], [25], [26], [27], [28], [29].

Sigmoidal functions have many important applications in neural networks [30]. For any neural net element, it uses a logistic function to produce the input signals. This function is often called activation function, see [31]. Three common instances of activations functions are the logistic function, the Hyperbolic Tangent Activation (HTA) function and the Half Hyperbolic Tangent Activation function which are given respectively by

$$\frac{1}{1+e^{-s}},\;\frac{e^{s}-e^{-t}}{e^{s}+e^{-s}},\;\frac{1-e^{-s}}{1+e^{-s}},$$

where s∈R. These functions were extensively studied in virtue of their nice properties. For example, they are univalent, differentiable and the mapping domain is a small range of outputs, see [32]. Joseph et al. [33] defined a modified sigmoid function and linked it with the class of univalent functions. Later, more subclasses associated with the sigmoid functions were introduced in a similar way. For example, in [34] the authors studied the class $S_{SG}^{⁎}$ in the definition of

$$S_{SG}^{⁎}=\left\{f\in A:\frac{zf^{\prime }(z)}{f(z)}≺\frac{2}{1+e^{-z}},\;z\in D\right\}.$$

Properties like radius estimates, coefficient bounds, growth and distortion theorem for this class were obtained.

Inspired by the above results, we now consider the classes which are associated with the activation functions defined by

$$J_{tanh}=\left\{f\in A:\frac{1+\frac{zf^{{}^{\prime \prime }}\left(z\right)}{f^{\prime }\left(z\right)}}{\frac{zf^{\prime }\left(z\right)}{f\left(z\right)}}≺1+\tanh\left(z\right),\;z\in D\right\},$$

and

$$J_{SG}=\left\{f\in A:\frac{1+\frac{zf^{{}^{\prime \prime }}\left(z\right)}{f^{\prime }\left(z\right)}}{\frac{zf^{\prime }\left(z\right)}{f\left(z\right)}}≺\frac{2}{1+e^{-z}},\;z\in D\right\}.$$

In this work, we aim to investigate the sharp bounds of some Hankel determinants for functions in the classes of $J_{tanh}$ and $J_{SG}$.

## 2 Preliminary results

The following lemmas are required to prove our key findings.

Lemma 1 *(see* [35], [36] *) Let* p∈P *and* $c_{1}\geq 0$ *. Then*

(2.1) $$2c_{2}=c_{1}^{2}+\xi \left(4-c_{1}^{2}\right),$$

(2.2) $$4c_{3}=c_{1}^{3}+2c_{1}\xi \left(4-c_{1}^{2}\right)-\left(4-c_{1}^{2}\right)\xi^{2}c_{1}+2\left(1-{\left|\xi \right|}^{2}\right)\left(4-c_{1}^{2}\right)\delta ,$$

(2.3) $$8c_{4}=c_{1}^{4}+\xi (4-c_{1}^{2})\left[4\xi +c_{1}^{2}\left(\xi^{2}+3-3\xi \right)\right]-4(4-c_{1}^{2})(1-{\left|\xi \right|}^{2})\cdot \left[\delta^{2}\bar{\xi }-\rho (1-{\left|\delta \right|}^{2})+c_{1}(\xi -1)\delta \right],$$

*for some* $\xi ,\delta ,\rho \in \bar{D}:=\left\{z\in C:\left|z\right|\leq 1\right\}$ *.*

Lemma 2 (see [37]) *Let*

$$Y\left(A,B,C\right)=\max_{z\in \bar{D}}\left\{\left|A+Bz+Cz^{2}\right|+1-{\left|z\right|}^{2}\right\}.$$

*If* AC≥0*, then*

$$Y\left(A,B,C\right)=\left\{ \begin{array}{cc} \left|A\right|+\left|B\right|+\left|C\right|,\; & if\;\left|B\right|\geq 2\left(1-\left|C\right|\right),\\ \;\\ 1+\left|A\right|+\frac{B^{2}}{4\left(1-\left|C\right|\right)},\; & if\;\left|B\right|<2\left(1-\left|C\right|\right), \end{array}\right.$$

*for some real numbers A, B and C.*

## 3 Main results

In view of

$$1+\tanh\left(z\right)=1+z-\frac{z^{3}}{3}+\frac{2}{15}z^{5}+\cdots ,\;z\in D$$

and

$$\frac{2}{1+e^{-z}}=1+\frac{1}{2}z-\frac{1}{24}z^{3}+\frac{1}{240}z^{5}+\cdots ,\;z\in D,$$

we take $\Psi (z)=1+B_{1}z+B_{2}z^{2}+B_{3}z^{3}+\cdots$ as a unified representation of univalent starlike function whose image domain of the unit disk D is a region in the right half plane. Suppose also that

$$J_{\Psi }=\left\{f\in A:\frac{1+\frac{zf^{{}^{\prime \prime }}\left(z\right)}{f^{\prime }\left(z\right)}}{\frac{zf^{\prime }\left(z\right)}{f\left(z\right)}}≺\Psi (z),\;z\in D\right\}.$$

Theorem 1 *Let*$B_{1}\geq \frac{1}{2}$*,*$B_{2}=B_{4}=0$*,*$B_{3}<0$*and*$f\in J_{\Psi }$*. Then*

$$\left|H_{2,2}\left(f\right)\right|\leq \left\{ \begin{array}{cc} \frac{1}{9}B_{1}\left|B_{3}\right|,\; & \left|B_{3}\right|\geq \frac{3B_{1}^{2}+4B_{1}}{8},\\ \frac{B_{1}^{2}\left(9B_{1}^{3}+24B_{1}^{2}+16B_{1}+36B_{3}\right)}{36\left(12B_{1}^{2}+7B_{1}+16B_{3}\right)},\; & \left|B_{3}\right|\leq \frac{3B_{1}^{2}+4B_{1}}{8}. \end{array}\right.$$

*This result is sharp. In the case of*$\left|B_{3}\right|\geq \frac{3B_{1}^{2}+4B_{1}}{8}$*, the extremal function*$f_{1}$*is given by the equation*

$$\frac{1+\frac{zf_{1}^{{}^{\prime \prime }}\left(z\right)}{f_{1}^{\prime }\left(z\right)}}{\frac{zf_{1}^{\prime }\left(z\right)}{f_{1}\left(z\right)}}=\Psi \left(z^{2}\right),\;z\in D.$$

*If*$\left|B_{3}\right|\leq \frac{3B_{1}^{2}+4B_{1}}{8}$*, we define*$f_{2}$*as*

$$\frac{1+\frac{zf_{2}^{{}^{\prime \prime }}\left(z\right)}{f_{2}^{\prime }\left(z\right)}}{\frac{zf_{2}^{\prime }\left(z\right)}{f_{2}\left(z\right)}}=\Psi \left(\omega_{2}(z)\right),\;z\in D,$$

*where*

$$\omega_{2}(z)=\frac{p_{2}(z)-1}{p_{2}(z)+1}$$

*and*

$$p_{2}(z)=\frac{1-z^{2}}{1+\sqrt{\frac{4B_{1}\left(6B_{1}-1\right)}{12B_{1}^{2}+7B_{1}+16B_{3}}}z+z^{2}},\;z\in D.$$

Proof Let $f\in J_{\Psi }$. Using subordination relationship, it is noted that

$$\frac{1+\frac{zf^{{}^{\prime \prime }}\left(z\right)}{f^{\prime }\left(z\right)}}{\frac{zf^{\prime }\left(z\right)}{f\left(z\right)}}≺\Psi (z),\;z\in D,$$

and thus there exists a Schwarz function *ω* satisfying that

$$\frac{1+\frac{zf^{{}^{\prime \prime }}\left(z\right)}{f^{\prime }\left(z\right)}}{\frac{zf^{\prime }\left(z\right)}{f\left(z\right)}}=\Psi \left(\omega \left(z\right)\right),\;z\in D.$$

Assume that $p\left(z\right)=\frac{1+\omega \left(z\right)}{1-\omega \left(z\right)}$ and

$$p\left(z\right)=1+c_{1}z+c_{2}z^{2}+c_{3}z^{3}+c_{4}z^{4}+\cdots ,\;z\in D.$$

Clearly, p∈P and

(3.1) $$\Psi \left(\omega \left(z\right)\right)=1+\frac{1}{2}B_{1}c_{1}z+\frac{1}{2}B_{1}\left(c_{2}-\frac{1}{2}c_{1}^{2}\right)z^{2}+\left[\frac{1}{8}\left(B_{1}+B_{3}\right)c_{1}^{3}-\frac{1}{2}B_{1}c_{1}c_{2}+\frac{1}{2}B_{1}c_{3}\right]z^{3}+\left[\frac{3}{8}\left(B_{1}+B_{3}\right)c_{1}^{2}c_{2}+\frac{1}{2}B_{1}c_{4}-\frac{1}{4}B_{1}c_{2}^{2}-\frac{1}{2}B_{1}c_{1}c_{3}-\frac{1}{16}\left(B_{1}+3B_{3}\right)c_{1}^{4}\right]z^{4}+\cdots .$$

Utilizing (1.1), we achieve

(3.2) $$\frac{1+\frac{zf^{{}^{\prime \prime }}\left(z\right)}{f^{\prime }\left(z\right)}}{\frac{zf^{\prime }\left(z\right)}{f\left(z\right)}}=1+a_{2}z+\left(-4a_{2}^{2}+4a_{3}\right)z^{2}+\left(-21a_{2}a_{3}+12a_{2}^{3}+9a_{4}\right)z^{3}+\left(80a_{2}^{2}a_{3}-32a_{2}^{4}-40a_{2}a_{4}-24a_{3}^{2}+16a_{5}\right)z^{4}+\cdots .$$

Combining the coefficients of (3.1) and (3.2), we obtain

(3.3) $$a_{2}=\frac{1}{2}B_{1}c_{1},$$

(3.4) $$a_{3}=\frac{1}{8}B_{1}c_{2}+\frac{1}{16}\left(4B_{1}^{2}-B_{1}\right)c_{1}^{2},$$

(3.5) $$a_{4}=\frac{1}{18}B_{1}c_{3}+\frac{1}{144}B_{1}\left(21B_{1}-8\right)c_{1}c_{2}+\frac{1}{288}\left(36B_{1}^{3}-21B_{1}^{2}+4B_{1}+4B_{3}\right)c_{1}^{3},$$

(3.6) $$a_{5}=\frac{1}{4608}\left(107B_{1}^{2}-54B_{3}-18B_{1}-276B_{1}^{3}+288B_{1}^{4}+80B_{1}B_{3}\right)c_{1}^{4}+\frac{1}{1152}\left(138B_{1}^{3}-107B_{1}^{2}+27B_{1}+27B_{3}\right)c_{1}^{2}c_{2}+\frac{1}{128}B_{1}\left(3B_{1}-2\right)c_{2}^{2}+\frac{1}{288}B_{1}\left(20B_{1}-9\right)c_{1}c_{3}+\frac{1}{32}B_{1}c_{4}.$$

Then it is got that

(3.7) $$a_{2}a_{4}-a_{3}^{2}=\frac{1}{2304}B_{1}\left(-12B_{1}^{2}+7B_{1}+16B_{3}\right)c_{1}^{4}+\frac{1}{576}B_{1}^{2}\left(6B_{1}-7\right)c_{1}^{2}c_{2}+\frac{1}{36}B_{1}^{2}c_{1}c_{3}-\frac{1}{64}B_{1}^{2}c_{2}^{2}.$$

Because $\left|H_{2,2}(f)\right|$ and the class $J_{\Psi }$ are invariant under rotations, we may assume $c_{1}:=c\in \left[0,2\right]$. Substituting (2.1) and (2.2) into (3.7), we get that

$$H_{2,2}\left(f\right)=\frac{1}{144}B_{1}B_{3}c^{4}+\frac{1}{192}B_{1}^{3}c^{2}\left(4-c^{2}\right)\xi -\frac{1}{2304}B_{1}^{2}\left(4-c^{2}\right)\left(36+7c^{2}\right)\xi^{2}+\frac{1}{72}B_{1}^{2}c\left(4-c^{2}\right)\left(1-{\left|\xi \right|}^{2}\right)\delta$$

for some $\xi ,\delta \in \bar{D}$. When c=0, we have

$$\left|H_{2,2}\left(f\right)\right|=\frac{1}{16}B_{1}^{2}\left|\xi^{2}\right|\leq \frac{1}{16}B_{1}^{2}.$$

When c=2, it is noted that $\left|H_{2,2}\left(f\right)\right|=\frac{1}{9}B_{1}\left|B_{3}\right|$. Now we assume that c∈(0,2). Then we find that

$$\left|H_{2,2}\left(f\right)\right|\leq \frac{1}{72}B_{1}^{2}c\left(4-c^{2}\right)\left(\left|\frac{B_{3}c^{3}}{2B_{1}\left(4-c^{2}\right)}+\frac{3}{8}B_{1}c\xi -\frac{36+7c^{2}}{32c}\xi^{2}\right|+1-{\left|\xi \right|}^{2}\right)=:\frac{1}{72}B_{1}^{2}c\left(4-c^{2}\right)R(A,B,C),$$

where

$$R(A,B,C)=\left|A+B\xi +C\xi^{2}\right|+1-{\left|\xi \right|}^{2}$$

and

$$A=\frac{B_{3}c^{3}}{2B_{1}\left(4-c^{2}\right)},\;B=\frac{3}{8}B_{1}c,\;C=-\frac{36+7c^{2}}{32c}.$$

Under the assumption that $B_{1}\geq \frac{1}{2}$ and $B_{3}<0$, we have AC>0. Hence, an application of Lemma 2 leads to the maximum value of *R*. Indeed, it is calculated that

$$\left|B\right|-2\left(1-\left|C\right|\right)=\frac{\left(6B_{1}+7\right)c^{2}-32c+36}{16c}.$$

In virtue of $B_{1}\geq \frac{1}{2}$, we see $\left(6B_{1}+7\right)c^{2}-32c+36\geq 0$ on (0,2). Then |B|≥2(1−|C|) and we find that

$$R(A,B,C)\leq \frac{\left(-12B_{1}^{2}-7B_{1}-16B_{3}\right)c^{4}+8B_{1}\left(6B_{1}-1\right)c^{2}+144B_{1}}{32B_{1}c\left(4-c^{2}\right)}.$$

Hence,

$$\left|H_{2,2}(f)\right|\leq \frac{B_{1}}{2304}\left[\left(-12B_{1}^{2}-7B_{1}-16B_{3}\right)c^{4}+8B_{1}\left(6B_{1}-1\right)c^{2}+144B_{1}\right]=:\frac{B_{1}}{2304}g\left(c^{2}\right),$$

where

$$g(t)=\left(-12B_{1}^{2}-7B_{1}-16B_{3}\right)t^{2}+8B_{1}\left(6B_{1}-1\right)t+144B_{1},\;t\in (0,4).$$

Let us set

$$D_{2}=-12B_{1}^{2}-7B_{1}-16B_{3},\;D_{1}=8B_{1}\left(6B_{1}-1\right),\;D_{0}=144B_{1}.$$

As we know,

$$\max_{0\leq t\leq 4}\left(D_{2}t^{2}+D_{1}t+D_{0}\right)=\left\{ \begin{array}{cc} D_{0},\; & D_{1}\leq 0,D_{2}\leq -\frac{D_{1}}{4},\\ \;\\ 16D_{2}+4D_{1}+D_{0},\; & D_{1}\geq 0,D_{2}\geq -\frac{D_{1}}{8}\\ \; & \text{or}\;D_{1}\leq 0,D_{2}\geq -\frac{D_{1}}{4},\\ \;\\ \frac{4D_{2}D_{0}-D_{1}^{2}}{4D_{2}},\; & D_{1}>0,D_{2}\leq -\frac{D_{1}}{8}. \end{array}\right.$$

From $6B_{1}^{2}-1\geq 0$, we have

$$\left|H_{2,2}(f)\right|\leq \left\{ \begin{array}{cc} \frac{B_{1}\left(16D_{2}+4D_{1}+D_{0}\right)}{2304},\; & D_{2}\geq -\frac{D_{1}}{8},\\ \frac{B_{1}\left(4D_{2}D_{0}-D_{1}^{2}\right)}{9216D_{2}},\; & D_{2}\leq -\frac{D_{1}}{8}. \end{array}\right.$$

After simplifications, we obtain the assertion in Theorem 1. □

Taking $B_{1}=1$, $B_{3}=-\frac{1}{3}$ and $B_{1}=\frac{1}{2}$, $B_{3}=-\frac{1}{24}$, we easily get the next two corollaries.

Corollary 1 *Suppose that*$f\in J_{tanh}$*. We have*

$$\left|H_{2,2}\left(f\right)\right|\leq \frac{37}{492}.$$

*The bound is sharp for the function*$f_{3}$*given by*

$$\frac{1+\frac{zf_{3}^{{}^{\prime \prime }}\left(z\right)}{f_{3}^{\prime }\left(z\right)}}{\frac{zf_{3}^{\prime }\left(z\right)}{f_{3}\left(z\right)}}=1+\tanh\left(\omega_{3}(z)\right),\;z\in D,$$

*where*

$$\omega_{3}(z)=\frac{p_{3}(z)-1}{p_{3}(z)+1}$$

*and*

$$p_{3}(z)=\frac{1-z^{2}}{1+\sqrt{\frac{60}{41}}z+z^{2}},\;z\in D.$$

*It can be also represented as*

$$f_{3}(z)=z-\frac{\sqrt{615}}{41}z^{2}+\frac{17}{82}z^{3}+\frac{65\sqrt{615}}{30258}z^{4}+\cdots ,\;z\in D.$$

Corollary 2 *Let* $f\in J_{SG}$ *. Then*

$$\left|H_{2,2}\left(f\right)\right|\leq \frac{109}{6720}.$$

*The equality is gained by the function* $f_{4}$ *given by*

$$\frac{1+\frac{zf_{4}^{{}^{\prime \prime }}\left(z\right)}{f_{4}^{\prime }\left(z\right)}}{\frac{zf_{4}^{\prime }\left(z\right)}{f_{4}\left(z\right)}}=\frac{2}{1+e^{-\omega_{4}(z)}},\;z\in D,$$

*where*

$$\omega_{4}(z)=\frac{p_{4}(z)-1}{p_{4}(z)+1}$$

*and*

$$p_{4}(z)=\frac{1-z^{2}}{1+\sqrt{\frac{24}{35}}z+z^{2}},\;z\in D.$$

*Its Taylor series expansion is*

$$f_{4}(z)=z-\frac{\sqrt{210}}{70}z^{2}-\frac{17}{280}z^{3}+\frac{737\sqrt{210}}{176400}z^{4}+\cdots ,\;z\in D.$$

Theorem 2 *Assume that* $f\in J_{tanh}$ *. Then*

$$\left|H_{3,1}\left(f\right)\right|\leq \frac{1}{81}.$$

*This result is best possible.*

Proof Let $f\in J_{tanh}$ and $f(z)=z+b_{2}z^{2}+b_{3}z^{3}+\cdots$. Replacing $B_{1}=1$ and $B_{3}=-\frac{1}{3}$ into (3.3), (3.4) and (3.5), we obtain

(3.8) $$b_{2}=\frac{1}{2}c_{1},$$

(3.9) $$b_{3}=\frac{1}{8}c_{2}+\frac{3}{16}c_{1}^{2},$$

(3.10) $$b_{4}=\frac{1}{18}c_{3}+\frac{13}{144}c_{1}c_{2}+\frac{53}{864}c_{1}^{3},$$

(3.11) $$b_{5}=\frac{1}{32}c_{4}+\frac{277}{13824}c_{1}^{4}+\frac{1}{128}c_{2}^{2}+\frac{11}{288}c_{1}c_{3}+\frac{49}{1152}c_{1}^{2}c_{2}.$$

For $f\in J_{tanh}$, it is calculated that

$$H_{3,1}\left(f\right)=2b_{2}b_{3}b_{4}-b_{2}^{2}b_{5}-b_{4}^{2}+b_{3}b_{5}-b_{3}^{3}.$$

From (3.8), (3.9), (3.10) and (3.11) along with $c_{1}=c\in \left[0,2\right]$, we have

$$H_{3,1}\left(f\right)=\frac{1}{5971968}(-629c^{6}+1086c^{4}c_{2}+7248c^{3}c_{3}-4932c^{2}c_{2}^{2}-11664c^{2}c_{4}+10080cc_{2}c_{3}-5832c_{2}^{3}+23328c_{2}c_{4}-18432c_{3}^{2}).$$

To simplify calculation, let $b=4-c^{2}$ in (2.1), (2.2) and (2.3). Now, from Lemma 1, we have

$$H_{3,1}\left(f\right)=\frac{1}{5971968}\{-128c^{6}+1536c^{3}b\left(1-{\left|\xi \right|}^{2}\right)\delta -768c^{4}\xi^{2}b-1134\xi^{2}b^{2}c^{2}+306\xi^{4}b^{2}c^{2}-864\xi b^{2}c\left(1-{\left|\xi \right|}^{2}\right)\delta -5832b^{2}{\left|\xi \right|}^{2}\left(1-{\left|\xi \right|}^{2}\right)\delta^{2}-1224\xi^{2}b^{2}c\left(1-{\left|\xi \right|}^{2}\right)\delta +144\xi bc^{4}-1026\xi^{3}b^{2}c^{2}-4608b^{2}{\left(1-{\left|\xi \right|}^{2}\right)}^{2}\delta^{2}-729b^{3}\xi^{3}+5832\xi^{3}b^{2}+5832\xi b^{2}\left(1-{\left|\xi \right|}^{2}\right)\left(1-{\left|\delta \right|}^{2}\right)\rho \}.$$

It is observed that

$$H_{3,1}\left(f\right)=\frac{1}{5971968}\left[\sigma_{1}\left(c,\xi \right)+\sigma_{2}\left(c,\xi \right)\delta +\sigma_{3}\left(c,\xi \right)\delta^{2}+\sigma_{4}\left(c,\xi ,\delta \right)\rho \right],$$

where

$$\sigma_{1}\left(c,\xi \right)=-128c^{6}+3\left(4-c^{2}\right)[102\left(4-c^{2}\right)c^{2}\xi^{4}+\left(4-c^{2}\right)\left(972-99c^{2}\right)\xi^{3}-2\left(756-61c^{2}\right)c^{2}\xi^{2}+48c^{4}\xi ],\sigma_{2}\left(c,\xi \right)=24\left(4-c^{2}\right)\left(1-{\left|\xi \right|}^{2}\right)c\left[64c^{2}-\left(4-c^{2}\right)\left(51\xi^{2}+36\xi \right)\right],\sigma_{3}\left(c,\xi \right)=72{\left(4-c^{2}\right)}^{2}\left(1-{\left|\xi \right|}^{2}\right)\left(-17{\left|\xi \right|}^{2}-64\right),\sigma_{4}\left(c,\xi ,\delta \right)=5832{\left(4-c^{2}\right)}^{2}\left(1-{\left|\delta \right|}^{2}\right)\left(1-{\left|\xi \right|}^{2}\right)\xi .$$

Taking |ξ|=γ and |δ|=y. In light of |ρ|≤1, we have

$$\left|H_{3,1}\left(f\right)\right|\leq \frac{1}{5971968}\left(\left|\sigma_{1}\left(c,\xi \right)\right|+\left|\sigma_{2}\left(c,\xi \right)\right|y+\left|\sigma_{3}\left(c,\xi \right)\right|y^{2}+\left|\sigma_{4}\left(c,\xi ,\delta \right)\right|\right)\leq \frac{1}{5971968}\left[\Theta \left(c,\gamma ,y\right)\right],$$

where

$$\Theta \left(c,\gamma ,y\right)=u_{1}\left(c,\gamma \right)+u_{2}\left(c,\gamma \right)y+u_{3}\left(c,\gamma \right)y^{2}+u_{4}\left(c,\gamma \right)\left(1-y^{2}\right),$$

and

$$u_{1}\left(c,\gamma \right)=128c^{6}+3\left(4-c^{2}\right)[102\left(4-c^{2}\right)c^{2}\gamma^{4}+\left(4-c^{2}\right)\left(972-99c^{2}\right)\gamma^{3}+2\left(756-61c^{2}\right)c^{2}\gamma^{2}+48c^{4}\gamma ],u_{2}\left(c,\gamma \right)=24\left(4-c^{2}\right)\left(1-\gamma^{2}\right)c\left[\left(4-c^{2}\right)\left(51\gamma^{2}+36\gamma \right)+64c^{2}\right],u_{3}\left(c,\gamma \right)=72{\left(4-c^{2}\right)}^{2}\left(1-\gamma^{2}\right)\left(17\gamma^{2}+64\right),u_{4}\left(c,\gamma \right)=5832{\left(4-c^{2}\right)}^{2}\left(1-\gamma^{2}\right)\gamma .$$

We are now to calculate the maximum value of Θ in the closed cuboid Ω:=[0,2]×[0,1]×[0,1]. Because

$$u_{3}(c,\gamma )-u_{4}(c,\gamma )=72{\left(4-c^{2}\right)}^{2}\left(1-\gamma^{2}\right)\left(17\gamma^{2}-81\gamma +64\right)\geq 0$$

on [0,2]×[0,1], we have

$$\frac{\partial \Theta }{\partial y}=u_{2}(c,\gamma )+2\left[u_{3}(c,\gamma )-u_{4}(c,\gamma )\right]y\geq u_{2}(c,\gamma )\geq 0.$$

Thus, we know Θ(c,γ,y)≤Θ(c,γ,1)=:W(c,γ). Note that

$$W\left(c,\gamma \right)=u_{1}(c,\gamma )+u_{2}(c,\gamma )+u_{3}(c,\gamma )=128c^{6}+3\left(4-c^{2}\right)\left[l_{4}(c)\gamma^{4}+l_{3}(c)\gamma^{3}+l_{2}(c)\gamma^{2}+l_{1}(c)\gamma +l_{0}(c)\right],$$

where

$$l_{4}(c)=102\left(4-c^{2}\right)\left(c^{2}-4c-4\right),l_{3}(c)=9{\left(2-c\right)}^{2}\left(11c^{2}+76c+108\right),l_{2}(c)=-2\left(61c^{4}+460c^{3}-1320c^{2}-816c+2256\right),l_{1}(c)=48c\left(c^{3}-6c^{2}+24\right),l_{0}(c)=512c^{3}-1536c^{2}+6144.$$

Then it reduces to get the maximum value of *W* on [0,2]×[0,1]. For c=2, we have W(2,γ)≡8192. If c=0, we obtain

$$W(0,\gamma )=-19584\gamma^{4}+46656\gamma^{3}-54144\gamma^{2}+73728=:\vartheta_{1}(\gamma ).$$

As

$$\vartheta_{1}^{\prime }\left(\gamma \right)=-78336\gamma^{3}+139968\gamma^{2}-108288\gamma \leq 0,\;\gamma \in (0,1),$$

we see $\vartheta_{1}$ have its maximum value 73728 at γ=0. When γ=0, we have

$$W(c,0)=128{\left(c^{3}-6c^{2}+24\right)}^{2}=:\vartheta_{2}(c).$$

Observing that $\vartheta_{2}^{\prime }\left(c\right)\leq 0$ on [0,2], we know $\vartheta_{2}$ attains its maximum value 73728 at c=0. Suppose that γ=1. Then

$$W(c,1)=359c^{6}-2580c^{4}-5040c^{2}+46656=:\vartheta_{3}(c).$$

From $\vartheta_{3}^{\prime }(c)=6c\left(359c^{4}-1720c^{2}-1680\right)\leq 0$ on [0,2], we have $\vartheta_{3}(c)\leq 46656$ with the maximum value achieved at c=0.

Now we aim to discuss the maximum value of *W* in (0,2)×(0,1). By solving the system of equations $\frac{\partial W}{\partial c}=0$ and $\frac{\partial W}{\partial \gamma }=0$, we find no critical points in (0,2)×(0,1). Indeed, the sixteen real critical points are listed as (0,0), (2,−2.6667), (−2,−0.9142), (−2,0.8204), (2.004,−5.2536), (−2.4708,−2.1319), (2.2070,−0.9670), (−1.4861,−0.8819), (3.9995,−0.0166), (2.3327,0.6385), (−0.4702,1.0504), (−2.2890,1.9931), (−2.0901,3.4220), (5.0642,0), (−1.7588,0) and (2.6946,0). Thus,

W(c,γ)≤73728,(c,γ)∈[0,2]×[0,1].

Then we can conclude that

Θ(c,γ,y)≤73728

on [0,2]×[0,1]×[0,1]. This leads to

$$\left|H_{3,1}\left(f\right)\right|\leq \frac{1}{5971968}\left[\Theta \left(c,\gamma ,y\right)\right]\leq \frac{1}{81}.$$

The bound is gained by $f_{5}$ taken as

$$\frac{1+\frac{zf_{5}^{{}^{\prime \prime }}\left(z\right)}{f_{5}^{\prime }\left(z\right)}}{\frac{zf_{5}^{\prime }\left(z\right)}{f_{5}\left(z\right)}}=1+\tanh\left(z^{3}\right),\;z\in D,$$

whose Taylor series expansion is

$$f_{5}(z)=z-\frac{1}{9}z^{4}+\cdots ,\;z\in D.$$

Theorem 2 has thus been proved. □

Theorem 3 *Assume that* $f\in J_{SG}$ *. Then*

$$\left|H_{3,1}\left(f\right)\right|\leq \frac{1}{324}.$$

*This bound is sharp.*

Proof Let $f\in J_{SG}$ and $f(z)=z+d_{2}z^{2}+d_{3}z^{3}+\cdots$. Substituting $B_{1}=\frac{1}{2}$ and $B_{3}=-\frac{1}{24}$ into (3.3), (3.4), (3.5) and (3.6), we find that

(3.12) $$d_{2}=\frac{1}{4}c_{1},$$

(3.13) $$d_{3}=\frac{1}{16}c_{2}+\frac{1}{32}c_{1}^{2},$$

(3.14) $$d_{4}=\frac{1}{36}c_{3}+\frac{5}{576}c_{1}c_{2}+\frac{13}{3456}c_{1}^{3},$$

(3.15) $$d_{5}=\frac{1}{64}c_{4}+\frac{11}{27648}c_{1}^{4}-\frac{1}{512}c_{2}^{2}+\frac{1}{576}c_{1}c_{3}+\frac{23}{9216}c_{1}^{2}c_{2}.$$

It is seen that

$$H_{3,1}\left(f\right)=2d_{2}d_{3}d_{4}-d_{2}^{2}d_{5}-d_{4}^{2}+d_{3}d_{5}-d_{3}^{3}$$

for $f\in J_{SG}$. From (3.12), (3.13), (3.14) and (3.15) along with $c_{1}=c\in \left[0,2\right]$, we deduce that

$$H_{3,1}\left(f\right)=\frac{1}{2985984}(5c^{6}-\frac{1155}{8}c^{4}c_{2}+510c^{3}c_{3}+\frac{279}{2}c^{2}c_{2}^{2}-1458c^{2}c_{4}+1476cc_{2}c_{3}-\frac{2187}{2}c_{2}^{3}+2916c_{2}c_{4}-2304c_{3}^{2}).$$

Let $b=4-c^{2}$ in (2.1), (2.2) and (2.3). Using Lemma 1, we get

$$H_{3,1}\left(f\right)=\frac{1}{2985984}\{-c^{6}+\frac{9}{4}c^{4}b\xi -24c^{4}b\xi^{2}-\frac{567}{16}c^{2}b^{2}\xi^{2}+\frac{153}{4}c^{2}b^{2}\xi^{4}-\frac{621}{4}c^{2}b^{2}\xi^{3}+729b^{2}\xi^{3}-\frac{2187}{16}b^{3}\xi^{3}-54cb^{2}\xi \left(1-{\left|\xi \right|}^{2}\right)\delta +48c^{3}b\left(1-{\left|\xi \right|}^{2}\right)\delta -729b^{2}{\left|\xi \right|}^{2}\left(1-{\left|\xi \right|}^{2}\right)\delta^{2}-153cb^{2}\xi^{2}\left(1-{\left|\xi \right|}^{2}\right)\delta -576b^{2}{\left(1-{\left|\xi \right|}^{2}\right)}^{2}\delta^{2}+729b^{2}\xi \left(1-{\left|\delta \right|}^{2}\right)\left(1-{\left|\xi \right|}^{2}\right)\rho \}.$$

Clearly, we can write

$$H_{3,1}\left(f\right)=\frac{1}{2985984}\left[\varsigma_{1}\left(c,\xi \right)+\varsigma_{2}\left(c,\xi \right)\delta +\varsigma_{3}\left(c,\xi \right)\delta^{2}+\varsigma_{4}\left(c,\xi ,\delta \right)\rho \right],$$

where

$$\varsigma_{1}\left(c,\xi \right)=-c^{6}+\frac{3}{16}\left(4-c^{2}\right)\xi [204\left(4-c^{2}\right)c^{2}\xi^{3}+9\left(4-c^{2}\right)\left(108-11c^{2}\right)\xi^{2}-\left(756-61c^{2}\right)c^{2}\xi +12c^{4}],\varsigma_{2}\left(c,\xi \right)=3\left(4-c^{2}\right)\left(1-{\left|\xi \right|}^{2}\right)c\left[16c^{2}-\left(4-c^{2}\right)\left(51\xi^{2}+18\xi \right)\right],\varsigma_{3}\left(c,\xi \right)=9{\left(4-c^{2}\right)}^{2}\left(1-{\left|\xi \right|}^{2}\right)\left(-17{\left|\xi \right|}^{2}-64\right),\varsigma_{4}\left(c,\xi ,\delta \right)=729{\left(4-c^{2}\right)}^{2}\left(1-{\left|\xi \right|}^{2}\right)\left(1-{\left|\delta \right|}^{2}\right)\xi .$$

Using |ρ|≤1 and taking |ξ|=γ, |δ|=y, we achieve

$$\left|H_{3,1}\left(f\right)\right|\leq \frac{1}{2985984}\left[\left|\varsigma_{1}\left(c,\xi \right)\right|+\left|\varsigma_{2}\left(c,\xi \right)\right|y+\left|\varsigma_{3}\left(c,\xi \right)\right|y^{2}+\left|\varsigma_{4}\left(c,\xi ,\delta \right)\right|\right],\leq \frac{1}{2985984}\left[Q\left(c,\gamma ,y\right)\right],$$

where

$$Q\left(c,\gamma ,y\right)=q_{1}\left(c,\gamma \right)+q_{2}\left(c,\gamma \right)y+q_{3}\left(c,\gamma \right)y^{2}+q_{4}\left(c,\gamma \right)\left(1-y^{2}\right),$$

with

$$q_{1}\left(c,\gamma \right)=c^{6}+\frac{3}{16}\left(4-c^{2}\right)[204\left(4-c^{2}\right)c^{2}\gamma^{4}+9\left(4-c^{2}\right)\left(108-11c^{2}\right)\gamma^{3}+\left(756-61c^{2}\right)c^{2}\gamma^{2}+12c^{4}\gamma ],q_{2}\left(c,\gamma \right)=3\left(4-c^{2}\right)\left(1-\gamma^{2}\right)c\left[\left(4-c^{2}\right)\left(51\gamma^{2}+18\gamma \right)+16c^{2}\right],q_{3}\left(c,\gamma \right)=9{\left(4-c^{2}\right)}^{2}\left(1-\gamma^{2}\right)\left(17\gamma^{2}+64\right),q_{4}\left(c,\gamma \right)=729{\left(4-c^{2}\right)}^{2}\left(1-\gamma^{2}\right)\gamma .$$

Now we are to maximize *Q* in the closed cuboid Π:=[0,2]×[0,1]×[0,1]. Since

$$q_{3}(c,\gamma )-q_{4}(c,\gamma )=9{\left(4-c^{2}\right)}^{2}{\left(1-\gamma \right)}^{2}\left(-17\gamma^{2}+47\gamma +64\right)\geq 0$$

on [0,2]×[0,1], we have

$$\frac{\partial Q}{\partial y}=q_{2}(c,\gamma )+2\left[q_{3}(c,\gamma )-q_{4}(c,\gamma )\right]y\geq q_{2}(c,\gamma )\geq 0.$$

Thus, Q(c,γ,y)≤Q(c,γ,1) for (c,γ)∈[0,2]×[0,1].

On y=1, ϒ(c,γ):=Q(c,γ,1) is given by

$$ϒ(c,\gamma )=q_{1}(c,\gamma )+q_{2}(c,\gamma )+q_{3}(c,\gamma )=c^{6}+\frac{3}{16}\left(4-c^{2}\right)\left[m_{4}(c)\gamma^{4}+m_{3}(c)\gamma^{3}+m_{2}(c)\gamma^{2}+m_{1}(c)\gamma +m_{0}(c)\right],$$

where

$$m_{4}(c)=204\left(4-c^{2}\right)\left(c^{2}-4c-4\right),m_{3}(c)=9{\left(2-c\right)}^{2}\left(11c^{2}+76c+108\right),m_{2}(c)=-\left(61c^{4}+1072c^{3}-3012c^{2}-3264c+9024\right),m_{1}(c)=12c\left(c^{3}-24c^{2}+96\right),m_{0}(c)=256c^{3}-3072c^{2}+12288.$$

By observing that $c^{2}-4c-4\leq 0$ for c∈[0,2], we know $m_{4}(c)\leq 0$. Also, it is clear that $m_{3}(c)\geq 0$ on [0,2]. Hence we have

$$ϒ(c,\gamma )\leq c^{6}+\frac{3}{16}\left(4-c^{2}\right)\left\{\left[m_{3}(c)+m_{2}(c)\right]\gamma^{2}+m_{1}(c)\gamma +m_{0}(c)\right\}=:V(c,\gamma ).$$

Now we aim to show that *V* attains its maximum value 9216 at (0,0) for (c,γ)∈[0,2]×[0,1]. Indeed, on the face c=2, we have V(2,γ)≡64. If c=0, then

$$V(0,\gamma )=9216-3852\gamma^{2}\leq 9216,\;\gamma \in [0,1].$$

When γ=0, we have

$$V(c,0)=c^{6}-48c^{5}+576c^{4}+192c^{3}-4608c^{2}+9216=:\iota_{1}(c).$$

Since $\iota_{1}^{\prime }(c)=0$ has no positive roots lie in (0,2), we get that $\iota_{1}$ achieves its maximum value 9216 at c=0. Suppose that γ=1. Then

$$V(c,1)=-\frac{67}{8}c^{6}+153c^{5}+\frac{1221}{4}c^{4}-1224c^{3}-2412c^{2}+2448c+5364=:\iota_{2}(c).$$

It is calculated that $\iota_{2}$ has only one positive root ${\overset{ˆ}{c}}_{0}\approx 0.4040$ lies in (0,2). Also, $\iota_{2}$ attains its maximum value about 5888.35 at $c={\overset{ˆ}{c}}_{0}$. Now it is time to obtain the maximum value of *V* in (0,2)×(0,1). By solving the system of equations $\frac{\partial V}{\partial c}=0$ and $\frac{\partial V}{\partial \gamma }=0$, we know there are no critical points lie in (0,2)×(0,1). Actually, the total fourteen real critical points are (0,0), (2,−9.3333), (−2,−0.7738), (−2,0.7269), (23.8310,0), (1.9243,0), (2.0934,0), (2.0000,−18.6691), (2.3182,−1.5488), (1.7069,−1.0516), (1.5966,−0.0240), (1.0921,1.1543), (2.1262,1.6766), (−2.0125,4.8784). Hence, V(c,γ)≤9216 on [0,2]×[0,1]. Thus, we deduce that

ϒ(c,γ)≤9216,(c,γ)∈[0,2]×[0,1].

Therefore,

Q(c,γ,y)≤9216,(c,γ,y)∈[0,2]×[0,1]×[0,1].

This leads to the conclusion that

$$\left|H_{3,1}\left(f\right)\right|\leq \frac{1}{2985984}\left[Q\left(c,\gamma ,y\right)\right]\leq \frac{1}{324}.$$

The bound is achieved by the function $f_{6}$ given by

$$\frac{1+\frac{zf_{6}^{{}^{\prime \prime }}\left(z\right)}{f_{6}^{\prime }\left(z\right)}}{\frac{zf_{6}^{\prime }\left(z\right)}{f_{6}\left(z\right)}}=\frac{2}{1+e^{-z^{3}}},$$

whose Taylor series expansion is

$$f_{6}(z)=z-\frac{1}{18}z^{4}+\cdots ,\;z\in D.$$

The assertion in Theorem 3 is thus proved. □

## Ethical approval

Not applicable.

**Funding**

Not applicable.

## CRediT authorship contribution statement

**YueJuan Sun:** Conceptualization, Funding acquisition. **Muhammad Arif:** Investigation, Writing – original draft. **Khalil Ullah:** Formal analysis, Investigation, Writing – review & editing. **Lei Shi:** Investigation, Supervision, Writing – review & editing. **Muhammad Imran Faisal:** Formal analysis, Supervision.

## Declaration of Competing Interest

The authors declare that they have no known competing financial interests or personal relationships that could have appeared to influence the work reported in this paper.

## Data Availability

Not applicable.
